# Psychological burden in family caregivers of patients with advanced cancer at initiation of specialist inpatient palliative care

**DOI:** 10.1186/s12904-019-0469-7

**Published:** 2019-11-18

**Authors:** Karin Oechsle, Anneke Ullrich, Gabriella Marx, Gesine Benze, Julia Heine, Lisa-Marie Dickel, Youyou Zhang, Feline Wowretzko, Kim Nikola Wendt, Friedemann Nauck, Carsten Bokemeyer, Corinna Bergelt

**Affiliations:** 1 Palliative Care Unit, Department of Oncology, Hematology and BMT, University Medical Center Hamburg-Eppendorf, Martinistr. 52, 20246 Hamburg, Germany; 2 Department of Medical Psychology, University Medical Center Hamburg-Eppendorf, Hamburg, Germany; 3 Department of Palliative Medicine, University Medical Center Goettingen, Goettingen, Germany; 4 Department of General Practice / Primary Care, University Medical Center Hamburg-Eppendorf, Hamburg, Germany

**Keywords:** Family caregiver, Cancer, Palliative care, Distress, Anxiety, Depression, Regression analysis

## Abstract

**Background:**

This study prospectively evaluated distress, depressive and anxiety symptoms as well as associated factors in family caregivers (FC) of advanced cancer patients at initiation of specialist inpatient palliative care.

**Methods:**

Within 72 h after the patient’s first admission, FCs were asked to complete German versions of the Distress Thermometer, Generalized Anxiety Disorder 7-item scale (GAD-7), Patient Health Questionnaire depression module 9-item scale (PHQ-9) for outcome measure. Multivariate logistic regression analyses were used to identify associated factors.

**Results:**

In 232 FCs (62% spouses/partners), mean level of distress was 7.9 (SD 1.8; range, 2–10) with 95% presenting clinically relevant distress levels. Most frequent problems were sadness (91%), sorrows (90%), anxiety (78%), exhaustion (77%) and sleep disturbances (73%). Prevalence rates of moderate to severe anxiety and depressive symptoms were 47 and 39%, respectively. Only 25% of FCs had used at least one source of support previously. In multivariate regression analysis, being female (OR 2.525), spouse/partner (OR 2.714), exhaustion (OR 10.267), and worse palliative care outcome ratings (OR 1.084) increased the likelihood for moderate to severe anxiety symptom levels. Being female (OR 3.302), low socio-economic status (OR 6.772), prior patient care other than home-based care (OR 0.399), exhaustion (OR 3.068), sleep disturbances (OR 4.183), and worse palliative care outcome ratings (OR 1.100) were associated with moderate to severe depressive symptom levels.

**Conclusions:**

FCs of patients presenting with indication for specialist palliative care suffer from high distress and relevant depressive and anxiety symptoms, indicating the high need of psychological support not only for patients, but also their FCs. Several socio-demographic and care-related risk-factors influence mental burden of FCs and should be in professional caregivers’ focus in daily clinical practice.

## Background

Regarding patients and their family caregivers (FC) as a “unit of care” is a basic principle of palliative care to improve the quality of life not only of the ill persons, but also of their families and friends [1]. FCs are important reference persons for the patients and have relevant impact on their wellbeing and quality of life, but at the same time, they are also affected by the patients' diseases with own burden and needs. Studies demonstrate various mental, social, physical, and economic burden in FCs [2, 3]. Psychosocial burden of FCs increases with the patients’ disease progression and, nearing death, it can even exceed the patients’ burden [4].

In previous studies, moderate or severe distress has been described in 55–90% of FCs assessed at different time points during the patients’ incurable cancer diseases [5–8]. Data on impact of sociodemographic characteristics, e.g. gender differences, are heterogeneous [5, 9], but psychosocial factors seem to be relevant including FCs’ self-care, role, stress, exhaustion, and overload [6, 10]. Further, mutual interactions between distress of patients and their families have been observed [9, 11].

Anxiety seems to be the most prevalent symptom in FCs, but the reported prevalence rates of significant anxiety vary largely between 32 and 72% [3, 5, 8, 12–15]. Prevalence rates for significant depression are lower in most previous studies, but also vary between 16 and 69% [3, 5, 8, 12–15]. Rumpold et al. described a decrease of anxiety prevalence over time from primary diagnosis of incurable cancer, whereas depressive symptoms remained stable [12]. FCs seem to be more likely to report anxiety symptoms, while depressive symptoms seem more frequent in patients [16, 17]. Dyads’ anxiety and depressive symptoms are positively associated [17]. FCs’ perceived hope, burden, resilience, coping strategies, self-care practices, nighttime sleep, physical activity, and pre-loss grief were associated with their mental burden, specifically with depressive symptoms [3, 12, 15, 18–20]. In addition, the patient’s coping strategy and acceptance of prognosis are also of impact on FCs’ depressive symptoms [17, 18]. While the FCs’ relationship to the patients influences FCs’ depressive symptoms [15], data on gender differences are controversial [5, 13].

Overall, previous studies evaluating mental burden of FCs and associated factors are inconsistent due to heterogeneous measures, evaluation of FCs of patients with different diseases, different assessment time points, and care settings.

Therefore, the aims of our study were to systematically evaluate distress, depressive and anxiety symptoms in the defined cohort of FCs of advanced cancer patients at the beginning of specialist inpatient palliative care (SIPC) on a palliative care ward. In addition, potential factors associated with mental burden including sociodemographic as well as disease- or care-related factors, prior utilization of psychosocial care, the FCs’ satisfaction with palliative care and perceived palliative care outcome were evaluated.

## Methods

### Study design

This prospective multicenter study was conducted in two University Medical Centers in Germany with similar structures for FCs support. Further, they represent two different regions of Germany with Hamburg representing a large urban city and Goettingen representing a smaller town in a more rural region.

During a 12-month period, FCs were consecutively assessed for study eligibility within 72 h after the patient’s first admission to the SIPC wards to represent the FCs’ situation prior to significant effects of SIPC. FCs inclusion criteria were being the primary informal caregiver as indicated by the patient – irrespective of biological or social relationship –, and being older than 18 years. Patients with advanced cancer were admitted to the SIPC wards due to significant physical and/or psychosocial symptoms prohibiting home care or care in non-specialist inpatient wards. Exclusion criteria were having only legal guardianship for the patient, inadequate language skills or insufficient cognitive function to complete the questionnaire according to the study personal’s assessment, and not being available during 72 h after the patient’s admission. Further, FCs of imminently dying patients were excluded.

FCs were contacted by trained study personal on the SIPC wards. Those who consented to participate received the questionnaire together with a return envelope. FCs who did not return the questionnaire within two working days were reminded once either by telephone or in person. In order to prevent potential study-induced burden, FCs were asked to immediately contact the palliative care teams in case of any problems or needs and a trained psycho-oncologist was on on-call demand.

Both ethics committees approved the study protocol (Hamburg PV5122; Goettingen 1/4/16). Written informed consent was obtained from all FCs prior to study inclusion.

### Measurements

FCs completed a set of questionnaires consisting of German versions of various standardized and mostly validated scales to measure distress, depressive and anxiety symptoms as outcome variables as well as satisfaction with palliative care and perceived palliative care outcome as potentially associated factors. Feasibility and acceptance of this large set of questionnaires in this sensible cohort of study participants has been studied in a 12-week pilot study [21].

The Distress Thermometer (DT) was used to measure subjective distress within the last week on an analogue scale rated from 0 “no distress” to 10 “extreme distress” [22, 23]. For the German version, a cut-off value of ≥5 reflects clinically relevant distress with need of professional psychological support [23]. The DT was also validated for distress screening in FCs revealing the same cut-off value [24]. Due to a high proportion of FCs exceeding this cut-off, we decided to also use a non-validated, study-specific cut-off of ≥8 to identify FCs with a range of problems that may reflect “severe distress” levels. The DT is accompanied by a problem list presenting specific concerns (rated “yes” or “no”) in the areas of practical, family, emotional, spiritual, and physical problems that might have caused distress. We used a shortened version adapted to relevant aspects for FCs with a reduced number of physical symptoms [21].

Generalized Anxiety Disorder 7-item scale (GAD-7) [25, 26] was used to assess anxiety symptoms. The 7 items assess the frequency of core symptoms of generalized anxiety disorder within the past two weeks. Items are scored on a four-point Likert scale rated from 0 “not at all” to 3 “nearly every day” with a total score ranging from 0 to 21. A score of 4 or less indicates the absence of anxiety symptoms, scores of 5–9 represent mild, 10–14 moderate and ≥ 15 severe anxiety symptom levels. For the German version of the GAD-7, age- and gender-adjusted data are available [26].

The Patient Health Questionnaire depression module 9-item scale (PHQ-9) [27, 28] was used to assess depressive symptoms. The 9 items assess the frequency of depressive symptoms within the past two weeks. Items are scored on a four-point Likert scale rated from 0 “not at all” to 3 “nearly every day” with a total score ranging from 0 to 27. A score of 4 or less indicates the absence of depressive symptoms, scores of 5–9 represent mild, 10–14 moderate and ≥ 15 severe depressive symptom levels. For the German version, age- and gender-adjusted data are available [28].

The Family Carer Satisfaction with Palliative Care scale (FAMCARE-2) consists of 17 items scored on a five-point Likert scale rated from “very dissatisfied “to “very satisfied” and a further category “not relevant”, with the latter being handled as missing data. The total score ranges from 17 to 85 with higher values indicating higher satisfaction [29, 30]. For FAMCARE-2 total score calculation, 20% of missing values were tolerated. We imputed the missing values by the mean score for the missing item [29]. Further, the FAMCARE-2 allows for calculation of four subscales: Symptom relief, Information, FC support and Patient’s psychosocial care.

The Integrated Palliative Care Outcome Scale (IPOS) combines the multidimensional evaluation for practical, emotional, and psychosocial concerns of the Palliative Care Outcome Scale (POS) [31–33] with detailed symptom assessment. Validation and factor structure of the IPOS staff proxy-report version have recently been published [33]. To assess the palliative care outcome as perceived by FCs, we adapted the 7-day recall version for staff, consisting of 17 items rated from 0 to 4 resulting in a total score from 0 to 68. Three subscale scores can be built: “Physical symptoms” (10 items), “Emotional symptoms” (4 items) and “Communication/practical issues” (3 items). Lower scores indicate better palliative care outcome. For IPOS total score calculation, 20% of missing values were tolerated. We imputed the missing values by the mean score for the missing items based on items completed by the individual.

In addition, various socio-demographic characteristics of the FCs, their relationship to and living situation with the patient, as well as FCs’ utilization of psychosocial support prior to SIPC were assessed. The socio-economic status was categorized using the “Winkler-Index”, which is an indicator-based approach used in the German National Health Survey [34, 35]. Migration status was assessed by a basic set of indicators for mapping migrant status of the German Federal Statistical Office [36]. Finally, the OSLO-3-Items-Social-Support-Scale was used to assess the perceived extent of informal social support [37–39]. The total score ranges from 3 to 14, with scores of 3–8 reflecting poor, 9–11 moderate and 12–14 strong support [39].

### Statistical analysis

We performed descriptive analyses to examine study population characteristics and to describe data from the measures used. We systematically compared female and male FCs with regard to their characteristics, distress, anxiety and depressive symptom levels, FCs’ satisfaction with palliative care, perceived palliative care outcome and utilization of psychosocial support services using chi-square-tests, Fisher’s exact test or two-sample t-tests (two-tailed). Further, we compared GAD-7 and PHQ-9 scores with German population-based samples using one-sample t-tests. For these analyses, we adjusted for age and gender by matching each FC with the value of a norm sample person from the same age and gender category.

To identify factors associated with high mental burden, bivariate statistics were calculated. Depending on the data’s level of measurement, statistically significant differences between groups (distress: cut-off < 8 and ≥ 8; depressive and anxiety symptoms: cut-off < 10 and ≥ 10) were assessed by either chi-square-tests or two-sample t-tests (two-tailed). Since we found only two variables to differ between distress groups, we decided to omit multivariate analyses for this outcome. For depressive and anxiety symptoms, multicollinearity was tested in all variables that revealed significant group differences in the bivariate analyses (spearman’s coefficient rho ≥0.6, tolerance values ≤0.6). Multivariate logistic regression analyses were modeled with moderate to severe depressive and anxiety symptoms being the dependent variables. FCs with none or mild symptoms were classified as reference groups, respectively. Within the regression analyses, we applied a backwards variable selection procedure (LR). Missing data was handled by list-wise deletion and the strengths of associations were expressed as odds ratios (OR) with 95% confidence intervals (CI).

All significance tests were two-tailed using a significance level of α < 0.05. Analyses were performed using SPSS software version 24.0 (IBM, USA).

## Results

### Family caregiver and patient characteristics

**Fig. 1:**
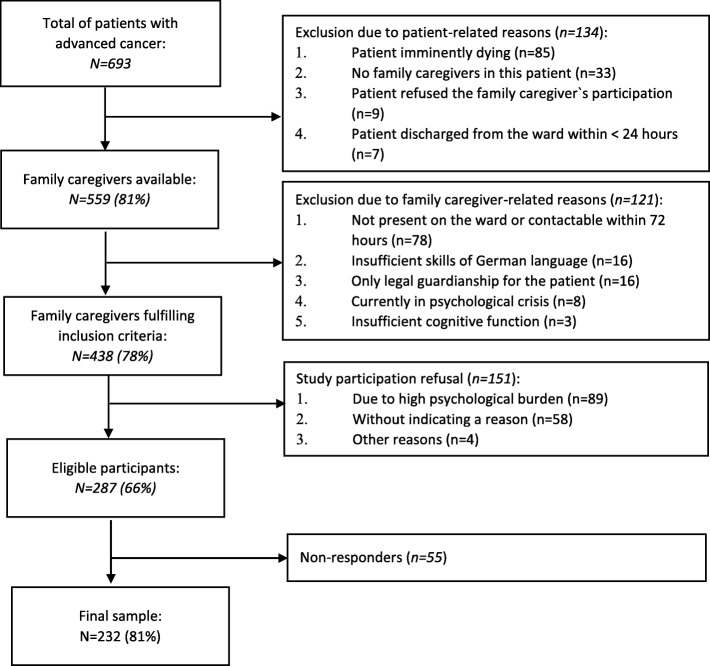
Study recruiting process and sample development

Between June 2016 and June 2017, 438 FCs matched the inclusion criteria, whereof 287 were willing to participate in this study (66%). Of these, 232 (81%) answered the questionnaires. A flow-chart of the recruiting process and sample development is presented in Fig. 1.

Median age of these 232 FCs was 55 years with 150 of them being female (65%) and 79 being male (35%). One-hundred eleven (48%) had a moderate socio-economic status, and only few were of migrant background (*n* = 20, 9%). FCs’ characteristics did not differ with regard to gender. For further details see Table 1.

**Table 1:** Family caregiver characteristics for the whole sample (*n* = 232) and separated for gender (*N* = 229)

		Whole sample (*N* = 232)	Family caregiver’s gender (*N* = 229)		
			Male (*n* = 79)	Female (*n* = 150)	*p*
Age (M, SD, Range)		55.5 (14.8); 20–88	54.7 (15.3); 20–86	55.8 (14.6); 21–88	.605 ^a^
Relationship to the patient. Patient is…	Spouse/partner	148 (63.8)	54 (68.4)	91 (60.7)	.073 ^b^
	Parent	61 (26.3)	20 (25.3)	41 (27.3)	
	Child	5 (2.2)	0 (0.0)	5 (3.3)	
	Sister/brother	7 (3.0)	0 (0.0)	7 (4.7)	
	Friend	5 (2.2)	1 (1.3)	4 (2.7)	
	Other	6 (2.6)	4 (5.1)	2 (1.3)	
Marital status	Single	36 (15.5)	14 (17.7)	22 (14.7)	.376 ^b^
	Married	164 (70.7)	58 (73.4)	104 (69.3)	
	Divorced	22 (9.5)	4 (5.1)	17 (12.0)	
	Widowed	7 (3.0)	2 (2.5)	5 (3.3)	
	Missing	3 (1.3)	1 (1.3)	1 (0.7)	
Having Children	Yes	164 (70.7)	52 (65.8)	111 (74.0)	.121 ^c^
	No	24 (27.6)	27 (34.2)	36 (24.0)	
	Missing	4 (1.7)	0 (0.0)	3 (2.0)	
Religious confession	Yes	153 (65.9)	48 (60.8)	103 (68.7)	.221 ^c^
	No	75 (32.3)	30 (38.0)	45 (30.0)	
	Missing	4 (1.7)	1 (1.3)	2 (1.3)	
Migrant background	None	212 (91.4)	71 (89.9)	139 (92.7)	.715 ^b^
	First generation	15 (6.5)	6 (7.6)	8 (5.3)	
	Second generation	5 (2.2)	2 (2.5)	3 (2.0)	
Educational level	Elementary school (≤ 9 years)	65 (28.0)	25 (31.6)	38 (25.3)	.469 ^c^
	Junior high school (10 years)	72 (31.0)	21 (26.6)	50 (33.3)	
	High school (12–13 years)	91 (39.2)	31 (39.2)	60 (40.0)	
	Missing	4 (1.7)	2 (2.5)	2 (1.3)	
Working situation	Currently employed	123 (53.0)	45 (57.0)	77 (51.3)	.222 ^c^
	Retired	73 (31.5)	25 (31.6)	46 (30.7)	
	Currently not employed for other reasons than retirement	26 (11.2)	5 (6.3)	21 (14.0)	
	Missing	10 (4.3)	4 (5.1)	6 (4.0)	
Socio-economic status	Low	44 (19.0)	13 (16.5)	29 (19.9)	.818 ^c^
	Middle	111 (47.8)	40 (50.6)	70 (46.7)	
	High	73 (31.5)	26 (32.9)	47 (31.3)	
	Missing	4 (1.7)	0 (0.0)	4 (2.7)	
Social support	Poor	43 (18.5)	15 (19.0)	27 (18.0)	.397 ^c^
	Moderate	90 (38.8)	35 (44.3)	54 (36.2)	
	Strong	98 (42.2)	29 (36.7)	68 (45.6)	
	Missing	1 (0.4)	0 (0.0)	1 (0.7)	

*Abbreviations*: *M* Mean, *SD* Standard deviation, *p* probability of type I error

^a^t-test, two-tailed

^b^Fisher’s exact test

^c^chi-square-test

The corresponding 232 patients were male in 53% (*n* = 118) and most of them were older than 60 years (*n* = 100, 67%). In 43% of patients (*n*=99), time between first cancer diagnosis and admission to the SIPC ward was less than 12 months. A patient decree had been prepared by 60% (*n* = 140) and a power of attorney by 69% (*n* = 159). Prior to admission, patients had lived at home without any nursing care service in 36% (*n* = 82), with a nursing service in 9% (*n* = 21), or with specialist outpatient palliative care service in 14% (*n* = 33). Other patients had been cared on another hospital inpatient ward in 36% (*n* = 82) or in a nursing home in 4% (*n* = 9).

### Distress

Mean level of distress was 7.9 (SD 1.8) out of 0–10 on the DT, and male and female FCs showed no significant differences. Clinically relevant distress with need of professional psychological support (cut-off value ≥5) was indicated in 95% and “severe distress” in 66% (Table 2). Out of 23 given problems, the five most frequently reported ones were sadness in 91% (*n* = 209), sorrows in 90% (*n* = 202), anxiety in 78% (*n* = 173), exhaustion in 77% (*n* = 171), and sleep disturbances in 73% (*n* = 163) of FCs.

**Table 2:** Results on depressive and anxiety symptoms, family caregiver satisfaction, palliative care outcome and utilization of psychosocial support services for the whole sample (*N* = 232) and separated for gender (*N* = 229)

	Whole sample (*N* = 232)	Family caregiver’s gender (*N* = 229)		
		Male (*n* = 79)	Female (*n* = 150)	*p*
Distress (DT)				
Total score (M, SD, Range)	7.9 (1.8); 0–10	7.7 (1.8); 3–10	8.0 (1.8); 2–10	.402 ^a^
Clinically relevant distress (n, %)				
Cut off < 5	11 (4.7)	5 (6.3)	6 (4.0)	.519 ^b^
Cut-off ≥ 5	221 (95.3)	74 (93.7)	144 (96.0)	
“Severe distress” (n, %)				
Cut off < 8	79 (34.1)	26 (32.9)	52 (34.7)	
Cut-off ≥ 8	153 (65.9)	53 (67.1)	98 (65.3)	
Anxiety symptoms (GAD-7)				
Total score (M, SD, Range)	9.4 (5.1); 0–21	8.1 (4.9); 0–20	10.0 (5.2); 0–21	**.008** ^a^
Symptom severity (n, %)				
None	48 (21.4)	22 (28.6)	26 (18.1)	**.032** ^c^
Mild	70 (31.3)	29 (37.7)	40 (27.8)	
Moderate	65 (29.0)	17 (22.1)	46 (31.9)	
Severe	41 (18.3)	9 (11.7)	32 (22.2)	
Depressive symptoms (PHQ-9)				
Total score (M, SD, Range)	9.0 (5.7); 0–27	7.4 (5.2); 0–23	9.9 (5.8); 0–27	**.001** ^a^
Symptom severity (n, %)				
None	49 (21.7)	24 (30.4)	25 (17.4)	**.008** ^c^
Mild	90 (39.8)	36 (45.6)	52 (36.1)	
Moderate	46 (20.4)	10 (12.7)	36 (25.0)	
Severe	41 (18.1)	9 (11.4)	31 (21.5)	
Family caregiver satisfaction (FAMCARE-2)				
Total score (M, SD, Range)	73.7 (9.6); 44–85	73.5 (9.9); 44–85	73.7 (9.5); 49–85	.919 ^a^
Subscales (M, SD, Range)				
Symptom relief	22.3 (2.9); 12–25	22.4 (2.9); 13–25	22.2 (2.9); 12–25	.658 ^a^
Information	16.5 (3.0); 7–20	16.5 (3.0); 7–20	16.5 (3.1); 10–20	.867 ^a^
Family caregiver support	17.0 (2.8); 8–20	16.9 (2.9); 8–20	17.0 (2.7); 10–20	.813 ^a^
Patient’s psychosocial care	17.9 (2.3); 10–20	17.7 (2.5); 10–20	17.9 (2.2); 11–20	.627 ^a^
Palliative care outcome (IPOS)				
Total score (M, SD, Range)	37.9 (7.7); 12–58	38.3 (6.9): 16–55	37.7 (8.3); 12–58	.595 ^a^
Subscales (M, SD, Range)				
Physical symptoms	22.2 (5.4); 6–36	22.5 (5.2); 6–36	22.1 (5.6); 7–36	.638 ^a^
Emotional symptoms	11.2 (2.9); 2–16	11.4 (2.6); 6–16	11.1 (2.0); 2–16	.424 ^a^
Communication/practical needs	4.1 (2.4); 0–11	4.1 (2.6); 0–11	4.1 (2.4): 0–11	.934 ^a^
Utilization of psychosocial support services				
Utilization of at least one source of information or support prior admission to the SIPC ward (n, %)				
Yes	58 (25.0)	14 (17.7)	43 (28.7)	.069 ^c^
No	174 (75.0)	65 (82.3)	107 (71.3)	
Barriers for service utilization (multiple answers possible) (n, % yes)				
Sufficient informal support (*N* = 196)	159 (81.1)	54 (79.4)	104 (81.9)	.674 ^c^
Missing subjective need (*N* = 189)	119 (63.0)	44 (64.7)	75 (62.0)	.710 ^c^
Missing time capacities (*N* = 181)	75 (41.4)	25 (36.8)	49 (43.8)	.356 ^c^
Preferring support by treating physicians (*N* = 181)	76 (42.0)	34 (52.3)	41 (35.7)	**.029** ^c^
No expectation of subjective benefit (*N* = 177)	62 (35.0)	27 (40.3)	34 (31.2)	.218 ^c^
No knowledge on psychosocial services (*N* = 188)	59 (31.4)	22 (32.4)	36 (30.3)	.765 ^c^
Services too far away (*N* = 177)	33 (18.6)	9 (13.4)	24 (21.8)	.165 ^c^
Potential burden to family/partnership (*N* = 182)	7 (3.8)	4 (6.0)	3 (2.6)	.426 ^b^
Attitudes towards psychosocial support services (n, %)				
Mainly/very positive	90 (38.8)	22 (27.8)	68 (45.3)	.073 ^b^
Rather positive	48 (20.7)	19 (24.1)	28 (18.7)	
Undecided	50 (21.6)	20 (25.3)	30 (20.0)	
Rather negative	12 (5.2)	7 (8.9)	5 (3.3)	
Very/mainly negative	16 (6.9)	7 (8.9)	8 (5.3)	

Significant group differences are marked in bold

*Abbreviations*: *M* Mean, *SD* Standard deviation, *p* probability of type I error, *DT* Distress Thermometer, *FAMCARE-2* Family Carer Satisfaction with Palliative Care scale, *GAD-7* Generalized Anxiety Disorder 7-item scale, *IPOS* Integrated Palliative Care Outcome Scale, *PHQ-9* Patient Health Questionnaire depression module 9-item scale, *SIPC* Specialist inpatient palliative care

^a^t-test, two-tailed

^b^Fisher’s exact test

^c^chi-square-test

### Anxiety and depressive symptoms

Prevalence of moderate to severe anxiety and depression were 47 and 39%, respectively. Absence of anxiety symptoms was prevalent in 21% and absence of depressive symptoms in 22% of FCs. We did find gender-specific differences with regard to anxiety and depressive symptom levels as well as GAD-7 or PHQ-9 total scores with females showing higher symptom levels (Table 2). Compared with age- and gender-adjusted German population [26, 28], levels of GAD-7 or PHQ-9 total scores were significantly higher in FCs (each *p* < .001).

### Family caregiver satisfaction and rating of palliative care outcome

FCs indicated high satisfaction with palliative care with a mean total FAMCARE-2 score of 73.7 (SD 9.6) of 17–85 possible points. Satisfaction was high in all four subscales with mean values of 22.3 (SD 2.9) of 5–25 possible points for Symptom relief, 16.5 (SD 3.0) of 5–20 points for Information, 17.0 (SD 2.8) of 5–20 for FC support, and 17.9 (SD 2.3) of 5–20 points for Patient’s psychosocial care. Across all scales, no gender differences emerged (Table 2).

FCs’ rated palliative care outcome at this early time point of SIPC with a mean total IPOS score of 37.9 (SD 7.7) of 68 possible points with lower ratings indicating worse outcome. Physical symptoms were rated with a mean score of 22.2 (SD 5.4) of 40 possible points, Emotional symptoms with 11.2 (SD 2.9) of 16 points and Communication/practical issues with 4.1 (SD 2.4) of 12 points. Again, no gender differences were observed (Table 2).

### Utilization of sources of information and support

Only 25% of FCs had used at least one source of information or support for their own problems and needs prior to the patient’s admission to the SIPC ward. This finding was not affected by gender (Table 2). Across all FCs, irrespective of utilization behavior, the three most common barriers for use of psychosocial services were sufficient informal support in 81%, missing subjective need in 63% and missing time capacities in 41%. Except for more males who more commonly reported preferring support by treating physicians, no gender differences were observed. Overall, 59% of FCs showed rather to very positive attitudes towards psychosocial support services, irrespective of their gender.

### Group comparisons for distress, depressive and anxiety symptom levels

Bivariate group comparisons revealed that FCs with “severe distress” (DT ≥8) significantly more often cared for younger patients (≤60 years) and reported higher satisfaction with palliative care (FAMCARE-2). Compared to those with none to mild anxiety symptoms, FCs with moderate to severe symptom levels (GAD-7 ≥ 10) were more often females, spouses/partners, reported lower monthly household net income as well as lower social support. Further, these FCs more frequently presented with exhaustion and sleep disturbances, and showed less favorable ratings of the palliative care outcome (IPOS). In comparison with FCs reporting none to mild depressive symptoms, those with moderate to severe symptom levels (PHQ-9 ≥ 10) were older (> 60 years), more often female, had low socio-economic status, and reported less social support. Further, home-based patient care was less common and ratings of the palliative care outcome (IPOS) were less favorable. While exhaustion was less frequent, sleep disturbances were a more common problem among these FCs (Table 3).

**Table 3:** Comparisons of family caregivers regarding levels of distress, depressive and anxiety symptoms

	Distress (*N* = 232)			Anxiety symptoms (*N* = 224)			Depressive symptoms (*N* = 226)		
	Below severe (DT < 8)	Severe (DT ≥ 8)		None/Mild (GAD-7 < 10)	Moderate/Severe (GAD-7 ≥ 10)		None/Mild (PHQ-9 < 10)	Moderate/Severe (PHQ-9 ≥ 10)	
	n (%)	n (%)	*p*	n (%)	n (%)	*p*	n (%)	n (%)	*p*
Family caregiver sociodemographic factors									
Age (M, SD)	55.3 (15.8)	55.1 (14.3)	.563 ^a^	55.0 (14.3)	54.6 (14.8)	.832 ^a^	56.4 (14.3)	52.6 (14.7)	.058 ^a^
Age categories									
≤ 60	45 (60.0)	95 (63.3)	.627 ^b^	72 (64.3)	68 (64.8)	.942 ^b^	78 (57.4)	62 (74.7)	**.010** ^b^
> 60	30 (40.0)	55 (36.7)		40 (35.7)	37 (35.2)		58 (42.6)	21 (25.3)	
Gender									
Male	26 (33.3)	53 (35.1)	.790 ^b^	51 (43.6)	26 (25.0)	**.004** ^b^	60 (43.8)	19 (22.1)	**.001** ^b^
Female	52 (66.7)	98 (64.9)		66 (56.4)	78 (75.0)		77 (56.2)	67 (77.9)	
Relationship to the patient									
Spouse/partner	46 (58.2)	98 (64.1)	.386 ^b^	65 (55.1)	73 (68.9)	**.034** ^b^	82 (59.0)	58 (66.7)	.248 ^b^
Other	33 (41.8)	55 (35.9)		53 (44.9)	33 (31.1)		57 (41.0)	29 (33.3)	
Having a partnership									
Yes	67 (84.8)	135 (88.8)	.383 ^b^	101 (86.3)	95 (89.6)	.451 ^b^	123 (88.5)	75 (87.2)	.774
No	12 (15.2)	17 (11.2)		16 (13.7)	11 (10.4)		16 (11.5)	11 (12.8)	
Having children									
Yes	57 (74.0)	107 (70.9)	.615 ^b^	76 (66.1)	80 (76.2)	.099 ^b^	95 (69.3)	64 (75.3)	.339 ^b^
No	20 (26.0)	44 (29.1)		39 (33.9)	25 (23.8)		42 (30.7)	21 (24.7)	
Religious confession									
Yes	53 (69.7)	100 (65.8)	.550 ^b^	77 (67.0)	68 (64.8)	.732 ^b^	92 (68.1)	55 (63.2)	.448 ^b^
No	23 (30.3)	52 (34.2)		38 (33.0)	37 (35.2)		43 (31.9)	32 (36.8)	
Educational level									
Elementary school (≤ 9 years)	22 (28.8)	43 (28.7)	.554 ^b^	30 (26.1)	33 (31.4)	.211 ^b^	36 (26.5)	28 (32.6)	.379 ^b^
Junior high school (10 years)	28 (35.9)	44 (29.3)		31 (27.0)	35 (33.3)		40 (29.4)	28 (32.6)	
High school (12–13 years)	28 (35.9)	63 (42.0)		54 (47.0)	37 (35.2)		60 (44.1)	30 (34.9)	
Working situation									
Working (full- or part-time)	44 (57.9)	86 (58.9)	.885 ^b^	71 (61.7)	58 (57.4)	.519 ^b^	78 (58.2)	52 (61.9)	.588 ^b^
Not working ^c^	32 (42.1)	60 (41.1)		44 (38.3)	43 (42.6)		56 (41.8)	32 (38.1)	
Monthly household net income									
< 2250 €	23 (31.9)	49 (35.0)	.775 ^b^	33 (29.5)	39 (40.6)	**.034** ^b^	40 (31.7)	83 (38.6)	.429 ^b^
2250€ - ≤ 4000€	30 (41.7)	60 (42.9)		45 (40.2)	42 (43.8)		53 (42.1)	35 (42.2)	
4000 € and more	19 (26.4)	31 (22.1)		34 (30.4)	15 (15.6)		33 (26.2)	16 (19.3)	
Socio-economic status									
Low	16 (20.3)	28 (18.8)	.434 ^b^	18 (15.3)	24 (23.5)	.113 ^b^	18 (13.2)	24 (27.9)	**.025** ^b^
Middle	42 (53.2)	69 (46.3)		55 (46.6)	51 (50.0)		71 (52.2)	37 (43.0)	
High	21 (26.6)	52 (34.9)		45 (38.1)	27 (26.5)		47 (34.6)	25 (29.1)	
Perceived social support									
Poor/moderate	41 (52.6)	92 (60.1)	.271 ^b^	60 (51.3)	69 (65.1)	**.037** ^b^	71 (51.4)	58 (66.7)	**.025** ^b^
Strong	37 (47.4)	61 (39.9)		57 (48.7)	37 (34.9)		67 (48.6)	29 (33.3)	
Patient characteristics									
Age									
≤ 60 years	16 (20.3)	59 (39.1)	**.004** ^b^	32 (27.4)	40 (38.1)	.088 ^b^	41 (29.9)	32 (36.8)	.286 ^b^
> 60 years	63 (79.7)	92 (60.9)		85 (72.6)	65 (61.9)		96 (70.1)	55 (63.2)	
Time between cancer diagnosis and admission to SIPC ward									
≤ 12 months	32 (42.7)	67 (45.0)	.744 ^b^	47 (41.2)	51 (50.0)	.196 ^b^	58 (43.0)	41 (49.4)	.354 ^b^
> 12 months	43 (57.3)	82 (55.0)		67 (58.8)	51 (50.0)		77 (57.0)	42 (50.6)	
Care-related aspects									
Care situation prior admission to SIPC ward									
At home	47 (60.3)	89 (58.9)	.848 ^b^	72 (62.1)	59 (56.2)	.374 ^b^	89 (64.5)	42 (49.4)	**.026** ^b^
Other	31 (39.7)	62 (41.1)		44 (37.9)	46 (43.8)		49 (35.5)	43 (50.6)	
Family caregiver cared for the patient prior admission to SIPC ward									
Yes	34 (44.7)	73 (49.0)	.545 ^b^	53 (45.7)	53 (51.0)	.435 ^b^	67 (50.4)	38 (44.2)	.429 ^b^
No	42 (55.3)	76 (51.0)		63 (54.3)	51 (49.0)		68 (49.6)	48 (55.8)	
Utilization of psychosocial counseling prior admission to SIPC ward									
Yes	16 (20.3)	42 (27.5)	.230 ^b^	26 (22.0)	32 (30.2)	.164 ^b^	29 (20.9)	28 (32.2)	.057 ^b^
No	63 (79.7)	111 (72.5)		92 (78.0)	74 (69.8)		110 (79.1)	59 (67.8)	
Family caregiver’s exhaustion ^d^									
Yes				71 (61.7)	96 (94.1)	**<.001** ^b^	42 (31.6)	9 (10.5)	**<.001** ^b^
No				44 (38.3)	6 (5.9)		91 (68.4)	77 (89.5)	
Family caregiver’s sleep disturbances ^d^									
Yes				73 (62.9)	87 (85.3)	**<.001** ^**b**^	85 (63.0)	75 (88.2)	**<.001** ^**b**^
No				43 (37.1)	15 (14.7)		50 (37.0)	10 (11.8)	
Family caregiver’s satisfaction with palliative care and perceived palliative care outcome									
Palliative care outcome (IPOS) - Total score (M, SD)	36.6 (8.3)	38.6 (7.4)	.088 ^a^	36.4 (7.8)	39.6 (7.4)	**.004** ^a^	36.9 (7.4)	39.8 (7.9)	**.008** ^a^
Satisfaction with palliative care (FAMCARE-2) - Total score (M, SD)	71.1 (10.6)	75.0 (8.8)	**.008** ^a^	73.0 (10.4)	74.3 (8.7)	.358 ^a^	73.5 (9.9)	73.7 (9.2)	.358 ^a^

Significant group differences are marked in bold

*Abbreviations*: *M* Mean, *SD* Standard deviation, *p* probability of type I error, *DT* Distress Thermometer, *FAMCARE-2* Family Carer Satisfaction with Palliative Care scale, *GAD-7* Generalized Anxiety Disorder 7-item scale, *IPOS* Integrated Palliative Care Outcome Scale, *PHQ-9* Patient Health Questionnaire depression module 9-item scale, *SIPC* Specialist inpatient palliative care

^a^t-test, two-tailed

^b^chi-square-test

^c^not working: retired, unemployed, housewife, in occupational training or studying

^d^two most frequently reported physical problems out of the adapted distress thermometer problem list. Not considered in group comparisons with distress as an outcome

### Factors associated with moderate to severe depressive and anxiety symptom levels

In the multivariate regression model, being female (OR 2.525), being a spouse/partner (OR 2.714), exhaustion (OR 10.267), and worse ratings of palliative care outcome (IPOS, OR 1.084) increased the likelihood for moderate to severe anxiety symptom levels. The regression model explained 32% of the total variance (Nagelkerke’s R^2^: 0.324). Significant factors associated with moderate to severe depressive symptom levels were being female (OR 3.302), low socio-economic status (OR 6.772), patient care other than home-based care prior to admission (OR 0.399), exhaustion (OR 3.068), sleep disturbances (OR 4.183), and worse ratings of the palliative care outcome (IPOS, OR 1.100). The regression model explained 40% of the total variance (Nagelkerke’s R^2^: 0.401; Table 4).

**Table 4:** Multivariate logistic regression analyses for moderate to severe depressive and anxiety symptom levels

	ß	SE	OR (95% CI)	*p*
Moderate/severe anxiety symptoms ^a^				
Family caregiver’s gender				
Male	Ref.			
Female	.926	.392	2.525 (1.171–5.445)	**.018**
Relationship to the patient				
Spouse/partner	.998	.395	2.714 (1.251–5.890)	**.012**
Other	Ref.			
Family caregiver’s exhaustion				
Yes	2.329	.582	10.267 (3.284–32.099)	**<.001**
No	Ref.			
Palliative care outcome (IPOS) - Total score	.081	.027	1.084 (1.303–1.147)	**.003**
Moderate/severe depressive symptoms ^b^				
Family caregiver’s gender				
Male	Ref.			
female	1.195	.428	3.302 (1.429–7.634)	**.005**
Socio-economic status				
Low	1.913	.589	6.772 (2.134–21.493)	**.001**
Middle	.196	.427	1.217 (.527–2.811)	.646
High	Ref.			
Social support				
Poor/moderate	.688	.397	1.989 (.914–4.327)	.083
Strong	Ref.			
Care situation prior admission to SIPC ward				
At home	−.920	.390	.399 (.186–.856)	**.018**
Other	Ref.			
Family caregiver’s exhaustion				
Yes	1.121	.561	3.068 (1.022–9.206)	**.046**
No	Ref.			
Family caregiver’s sleep disturbances				
Yes	1.431	.510	4.183 (1.541–11.358)	**.005**
No	Ref.			
Palliative care outcome (IPOS) - Total score	.095	1.524	1.100 (1.039–1.164)	**.001**

*Abbreviations*: *ß* unstandardized regression coefficient, *SE* standard error, *OR* odds ratio for independent variables, *CI* 95% confidence interval, *p* probability of type I error, *Ref*. Reference group, *SIPC* specialist inpatient palliative care, *IPOS* Integrated Palliative Care Outcome Scale

^a^Reference group: none to mild anxiety symptoms (GAD-7 < 10); due to missing values, 168 out of 232 family caregivers were included into the final regression model; potentially associated factors included in the regression model at step 1: gender, household net income, relationship to the patient, social support, exhaustion, sleep disturbances, IPOS total score; tolerance values between .790 and .976

^b^Reference group: none to mild depressive symptoms (PHQ-9 < 10); due to missing values, 174 out of 232 family caregivers were included into the final regression model; potentially associated factors included in the regression model at step 1: age groups, gender, socio-economic status, social support, care situation prior admission to SIPC ward, exhaustion, sleep disturbances, IPOS total score; tolerance values between .769 and .971

Factors significantly associated with the outcome variable are marked in bold

## Discussion

This study evaluated mental burden, including distress, depressive and anxiety symptoms in FCs of patients with advanced cancer at initiation of SIPC, thus also representing the FCs’ situation prior to first contact with the SIPC ward. Our data on the prior care setting suggests that for the majority of 86% patients it was the first contact with any kind of specialist palliative care.

Clinically relevant distress was observed in almost all FCs (95%) in this study. Previous studies have reported moderate or severe distress varying from 55 to 90% of FCs at different time points during the patients’ incurable cancer diseases, measured with different assessment instruments [5–8]. Areia et al. reported almost identical rates of severe psychological distress assessed by the Brief Symptom Inventory among FCs of patients with terminal cancer [8], while Rosenberger et al. found similarity high rates (90%) of relevant distress (DT ≥ 5) in FCs of cancer patients across all cancer stages [5]. In FCs of patients with high-grade glioblastoma, distress (DT ≥5) was most prominent proximal to diagnosis with 62%, but remained high during 3 (61%) and 6 months follow-up (58%) [7]. Our assessment included a modified DT problem list, demonstrating that the five most distressing problems in FCs were sadness, sorrows, anxiety, exhaustion, and sleep disturbances.

Regarding socio-demographic and patient-related characteristics, FCs with “severe distress”, which we defined at a cut-off of DT ≥ 8, significantly more often cared for younger patients, while previous studies did not report patient age but FCs’ age as predictor of FCs’ burden [8]. Two studies have revealed the relationship to the patient as significant associated factors [8, 9], which could not be confirmed in our study. The role of FCs’ gender was heterogeneous in previous studies [5, 8, 9], but no such effect was observed in our analysis. Satisfaction with palliative care was higher in FCs with “severe distress”. Care satisfaction has not been evaluated as factor potentially associated with mental burden, but the satisfaction of needs by healthcare professionals [8], managing patients’ psychological symptoms [40, 41], and the construct “exhaustion and overload” [10] have previously been described as impact factors. We assume that high satisfaction with care on the SIPC ward might be influenced by FCs relief from caregiving problems and overload. Thus, palliative care on the SIPC ward might be overestimated by FCs’ in terms of downward-comparison to the care situation prior to initiation of SIPC. In contrast, ratings of palliative care outcome including more objective parameters of the patient’s situation was rather moderate or low in all subscales (Physical symptoms, Emotional symptoms, Communication/practical issues) without any association with FCs’ distress.

Moderate to severe anxiety and depressive symptoms were observed in 47 and 39% of FCs at admission to SIPC, respectively, and mean scores of symptom levels were significantly higher than those of an age- and gender-adjusted population sample [26, 28]. These results are in line with previous studies reporting prevalence rates of significant anxiety between 32 and 72% at different time points of the advanced cancer patients’ disease trajectories [3, 5, 8, 10, 12, 13, 15]. Prevalence rates of significant depression were also lower in most previous studies but also with large variations of 16–69% [3, 5, 8, 10, 12, 13, 15]. In our study, one possible explanation for lower rates of significant depression could be that FCs of patients, who qualified for referral to SIPC, have been encountered with numerous stressors within the last days, which specifically may give rise to anxiety than depression. Both questionnaires used to assess symptoms of anxiety and depression, the GAD-7 and PHQ-9, refer to FCs symptoms within the last 2 weeks.

Our analysis of correlates of moderate to severe depressive and anxiety symptoms revealed several socio-demographic factors to be associated with elevated mental burden. Female FCs had a higher risk for moderate to severe anxiety and depressive symptom levels, supporting the results of Grande et al. who reported similar findings [42]. While in the study of Grande et al. differences in mental burden were more pronounced in younger FCs [42], in our study older FC age was significantly associated with moderate to severe depressive levels in bivariate analyses, but this effects disappeared in multivariate analyses. Moderate to severe anxiety symptoms, but not depressive symptoms, were more frequent in spouses or partners compared to other kinds of relationship to the patient. In contrast, studies evaluating FCs of advanced cancer patients over all stages of disease showed significantly more symptoms of both, anxiety and depression, in spouses [12, 43] and Nielsen et al. reported highest levels of depressive symptoms in bereaved partners [15]. Lower monthly household net income was associated with moderate to severe anxiety symptoms, and in multivariate analyses, low socio-economic status showed to be an important risk factor for moderate to severe depressive symptoms. These findings demonstrate the high relevance of socio-economic aspects when seeking to address FCs mental burden.

Considering care-related aspects, in multivariate analyses, patient care in the home care setting prior SIPC was associated with less depressive symptoms. This might suggest that FCs’ feelings that the patient has been cared for at the place according to his or her wishes might be a protective factor. Sleep disturbances were more frequent in FCs with moderate to severe anxiety and depressive symptoms in our study. This has also been described by Peak et al. who found an association between short nighttime sleep and FCs’ anxiety and depressive symptoms [20]. Nevertheless, it remains unclear whether sleep disturbances cause anxiety and depressive symptoms or vice versa. Further, exhaustion was more prevalent in FCs with anxiety and depressive symptoms, but the association with anxiety symptoms disappeared in multivariate analysis. Several previous studies suggested associations between self-care practices and the FCs’ own physical capacities, which might relevantly contribute to FCs’ exhaustion, with prevalence of FCs’ psychological burden [12, 18, 20, 43]. However, FCs’ depressive – not anxiety – symptoms seem to be more strongly related to their resilience and overall burden [19, 20, 44]. Regarding FCs assessment of patient care and palliative care outcome, satisfaction with palliative care did not show any association with mental burden. However, worse perceptions of palliative care outcome, which includes FCs’ estimation of patient’s physical symptoms, emotional symptoms as well as communication/practical needs, was associated with moderate to severe depressive and anxiety symptoms. Oechsle et al. [13] demonstrated a higher risk of overestimation of patient’s symptom burden among FCs with higher levels of depressive and anxiety symptoms. In aggregate, these findings emphasize the importance of understanding the FCs perspective on the patient’s situation when addressing FCs mental burden.

We found no association between having utilized psychosocial support services prior SIPC and mental burden. But, interestingly, only one fourth of FCs in our study had used professional psychosocial support, and the most frequent reason for non-use was sufficient informal support. Previous studies also reported low rates of psychosocial service use in FCs of about 10–30% despite of partly high interest in support or high psychological symptom burden [43, 45, 46]. This raises the question whether FCs are able to estimate and appreciate their own psychosocial needs in this difficult situation or if they prefer informal support due to lacking support offers adapted to their specific situation. Some first interventional studies have evaluated different psychological interventions as stand-alone interventions or part of integrated SIPC [47–53], but results were heterogeneous with positive effects only on parts of outcome parameters or without sustainability.

This study was the first prospectively evaluating mental burden in a large cohort of FCs of advanced inpatient cancer patients at the time of admission to SIPC at two university centers representing different regions. Internationally, most FC-directed palliative care research focuses on the setting of home-based care, but FCs’ burden in the SIPC setting has been less investigated, although circumstances qualifying the patient for SIPC might impact FC burden and specific support might be required. Further, only a limited body of palliative care research in German-speaking countries focuses on the FCs’ situation. However, the culture of caregiving and characteristics of health care systems may influence FCs’ mental burden.

Yet, it is notable that the results of our study are subject to certain limitations. Among those, generalization of results was most relevant. Due to our study design, results reflect the situation of FCs at initiation of specialist palliative care in an inpatient setting and might not be directly transferred to an outpatient palliative care setting. Referral of cancer patients to SIPC is often initiated late in the disease trajectory or close to death, often associated with high and complex symptom burden [54]. Thus, generalization should be applied with awareness for possible bias towards higher distress in FCs of patients admitted to SIPC. Though high psychological burden had not been defined as exclusion criterion a priori, 89 FCs refused study participation for this reason. This may affect generalization, as FCs included may be burdened to a lesser extent or may represent more extreme positive or negative experience, which may have motivated study participation. Further, data on FCs satisfaction with palliative care (FAMCARE-2) should not be taken as representative for the experiences of FCs during or at the end of SIPC, as data was obtained within 72 h after the patient had been admitted. It is a novelty that an adapted version of the staff proxy-report version of the Integrated Palliative Care Outcome Scale (IPOS) was used in this study. Although the IPOS was found to be a valid and reliable tool [33], psychometric properties including reliability may not apply to proxy-reports of FCs. Specific prerequisites of acting as FC, such as direct personal involvement and being a lay person in most cases, may influence the assessment, and data has to be interpreted with awareness of these limiting factors. Nevertheless, with exception of the IPOS, we used valid and reliable measures for the assessment of distress, anxiety and depressive symptoms as well as variables assumed to be associated with mental burden. Finally, we focused on FCs’ situation, thus not including potential effects of the patient’s psychological distress, coping strategies or prognostic understanding on FCs’ mental burden. Nevertheless, prior studies have underscored the importance of these risk factors for elevated burden among FCs [16, 17].

## Conclusions

FCs of patients admitted to SIPC suffer from high psychological distress, and relevant anxiety and depressive symptoms. This demonstrates the high need of psychological support as central part of specialist inpatient palliative care not only for the patients, but also for their FCs. Several socio-demographic and care-related factors are significantly associated with FCs’ mental burden and should be in professional carers’ focus in daily clinical practice. FCs’ female gender, exhaustion and worse ratings of the patient’s palliative care outcome seem to be associated with both moderate to severe anxiety and depressive symptoms. Further supportive or psychosocial interventions - even at an earlier stage of the disease - should be developed and evaluated to better address FCs’ problems and psychosocial needs in future. Since studies underscore the co-occurrence and interdependence of FCs and patients mental burden, targeting FCs problems via such interventions might have the potential to also enhance patient’s quality of life and mental well-being. However, interrelationships of mental burden in dyads of patients and their FCs also raise the question, when individual versus dyadic interventions would be most beneficial. Therefore, future research on FCs mental burden and evaluation of interventions designed to address these burden should also include assessment of the patient’s psychological distress.

## Data Availability

The authors have full control over the primary data. The data are analyzed in this study are housed at the Palliative Care Unit, Department of Oncology, Hematology and BMT, University Medical Center Hamburg-Eppendorf, Martinistrasse 52, 20246 Hamburg, Germany. As per the ethical committee approval, this dataset is subject to ethical restrictions, and informed written consent of study participants does not include publication of raw data or disclosure to third parties. All relevant data for the conclusions are presented in the manuscript.

## Abbreviations

CI: Confidence interval
DT: Distress Thermometer
FAMCARE-2: Family Carer Satisfaction with Palliative Care scale
FC: Family caregiver
GAD-7: Generalized Anxiety Disorder 7-item scale
IPOS: Integrated Palliative Care Outcome Scale
OR: Odds ratio
PHQ-9: Patient Health Questionnaire depression module 9-item scale
SIPC: Specialist inpatient palliative care
